# Indirect detector for ultra-high-speed X-ray micro-imaging with increased sensitivity to near-ultraviolet scintillator emission

**DOI:** 10.1107/S1600577524007306

**Published:** 2024-08-28

**Authors:** Bratislav Lukić, Alexander Rack, Lukas Helfen, Daniel J. Foster, Alexey Ershov, Richard Welss, Stéphane François, Xavier Rochet

**Affiliations:** a ESRF – The European Synchrotron, 38043Grenoble Cedex 9, France; b Henry Royce Institute, Department of Materials The University of Manchester Manchester United Kingdom; c Karlsruhe Institut of Technology 76034Eggenstein-Leopoldshafen Germany; d Institut Laue-Langevin 38042Grenoble Cedex 9 France; e Professorship for Fluid Systems Technology Friedrich-Alexander-Universität Erlangen-Nürnberg 91058Erlangen Germany; f Optique Peter, 69210 Lentilly, France; Australian Synchrotron, Australia

**Keywords:** indirect X-ray detectors, high-speed imaging, micro-imaging, polychromatic radiation

## Abstract

Ultra-high-speed synchrotron-based hard X-ray (*i.e.* above 10 keV) imaging is gaining a growing interest in a number of scientific domains for tracking non-repeatable dynamic phenomena at spatio-temporal microscales. This work describes an optimized indirect X-ray imaging microscope designed to achieve high performance at micrometre pixel size and megahertz acquisition speed. The entire detector optical arrangement has an improved sensitivity within the near-ultraviolet (NUV) part of the emitted spectrum (*i.e.* 310–430 nm wavelength). When combined with a single-crystal fast-decay scintillator, such as LYSO:Ce (Lu_2−*x*_Y_*x*_SiO_5_:Ce), it exploits the potential of the NUV light-emitting scintillators. The indirect arrangement of the detector makes it suitable for high-dose applications that require high-energy illumination. This allows for synchrotron single-bunch hard X-ray imaging to be performed with improved true spatial resolution, as herein exemplified through pulsed wire explosion and superheated near-nozzle gasoline injection experiments at a pixel size of 3.2 µm, acquisition rates up to 1.4 MHz and effective exposure time down to 60 ps.

## Abstract 2

This study introduces an optimized indirect X-ray microscope capable of achieving micrometre pixel size and megahertz acquisition speed, leveraging enhanced sensitivity in the near-ultraviolet spectrum and single-crystal fast-decay scintillators. This development enables high-resolution imaging for dynamic phenomena, exemplified by experiments with pulsed wire explosion and superheated near-nozzle gasoline injection.

## 1. Introduction

Exploiting the pulsed nature of synchrotron radiation sources has led to an increased interest in time-resolved imaging studies of probing materials under dynamic transient phenomena of a non-repetitive nature (Wang *et al.*, 2023 ▸). Optically opaque solids, as well as optically obscured fluids, can be studied in full field at spatio-temporal microscales under extreme conditions, *in situ*. These span over shock loading (Escauriza *et al.*, 2020 ▸; Pradel *et al.*, 2022 ▸), high loading rates (Hudspeth *et al.*, 2013 ▸; Lukić *et al.*, 2024 ▸; Zielinski *et al.*, 2023 ▸), turbulent flow jetting (Wang *et al.*, 2008 ▸; Lee *et al.*, 2011 ▸), cavitation bubble dynamics and coalescence (Fezzaa & Wang, 2008 ▸; Bokman *et al.*, 2023 ▸), as well as hydrodynamic instabilities at shock-accelerated interfaces during fusion-relevant scenarios (Maler *et al.*, 2022 ▸; Strucka *et al.*, 2023 ▸; Montgomery, 2023 ▸), to mention a few. Aside from intrinsically occurring on microsecond scales (*e.g.* crack propagation and fragmentation of solids), many of these applications often require additional environments (*i.e.* opaque sample holders, confinement or vacuum chambers, high-temperature furnaces, cryogenic or host fluid environments), which make the phenomena of interest difficult to probe. Hard X-rays, such as those emitted by third- and fourth-generation synchrotron storage ring facilities (Raimondi *et al.*, 2023 ▸), provide a unique opportunity to access through-volume information at high spatio-temporal scales owing to high brilliance and high photon energy (*i.e.* above 10 keV) (Rack, 2020 ▸). Under such extreme conditions, an adequate imaging measurement able to well discretize the phenomena is the result of an interaction with a small integer number (*i.e.* multi-bunch or single bunch) X-ray pulse. While the temporal resolution in the former is dictated by the detector shutter speed (of the order of several 10 ns), in the latter, the ultimate temporal resolution depends on the storage ring radio frequency and single-bunch duration (of the order of several 10 ps). In both situations, the recorded radiograph is a result of the X-ray pulse(s) of a single or small integer number of bunches impinging the detector, consequently resulting in a limited photon count that scales with the maximum current that a stable single electron bunch in the storage ring can carry, as well as the detector spatial sampling. Therefore, a suitable imaging detector needs to have sufficient sensitivity to capture a recording of multiple consecutive frames in order to directly observe potentially unique dynamics occurring at the micrometre and microsecond scales, with the ability to map the entire temporal response of a sample, since phenomena of interest can be inherently unpredictable.

In this article, an optimized indirect detector near-ultraviolet (NUV) sensitive X-ray microscope, developed and designed by Optique Peter (France), is presented, with the optical components [*i.e* the objective lens, the reflective mirror and the tube lens (Douissard *et al.*, 2012 ▸)] matched to the emission spectra of an LYSO:Ce scintillator. The detector enables reaching of 10× magnification with higher efficiency (up to four times) compared with conventional microscopy tandems, allowing ultra-fast single-bunch microscopy with white beam radiation to be achieved with unprecedented spatio-temporal resolution.

## 2. State of the art

Historically, the developments of (ultra)fast area X-ray detectors have been driven by X-ray imaging and X-ray diffraction measurements. The introduction of direct pixelated array detectors (PADs) as a combination of Si-based diode and charge-coupled device imagers on a pixel-to-pixel base alleviated the time-sampling limits of using mechanical X-ray chopper systems to isolate single or multiple X-ray bunches from the pulsed source (Cammarata *et al.*, 2009 ▸). The use of PADs also stimulated the detection schemes towards exploiting the natural timing modes of the synchrotron and pulsed operation of free electron laser (FEL) X-ray radiation sources (Barna *et al.*, 1997 ▸; Gruner *et al.*, 2002 ▸). The high charge sensitivity of these devices has made them suitable due to their high detection efficiency, especially in low-signal applications such as in ultra-fast diffraction imaging (van Driel *et al.*, 2020 ▸). Further advancement to hybrid complementary metal-oxide-semiconductor technologies allowed reaching of sub-nanosecond exposure times with a relatively large pixel count per acquired frame (Allahgholi *et al.*, 2015 ▸; Philipp *et al.*, 2016 ▸). Single-shot exposure detectors allowed for reaching of the unprecedented temporal resolution of the order of femtoseconds by isolating single-bunch structures emitted by X-ray FELs (Katagiri *et al.*, 2023 ▸; Schropp *et al.*, 2015 ▸). However, the penetration depth stayed limited due to low peak energies (*i.e.* below 10 keV). Some recent developments focused on GaAs-based detectors leading to a decreased pixel size with higher sensitivity to X-rays in the 10–20 keV range, extending the direct detector possibilities to in-line phase-contrast imaging within ultra-fast X-ray holography with nanosecond sampling (Hart *et al.*, 2019 ▸; Hodge *et al.*, 2022 ▸). Despite the efforts in detector developments with higher *Z* number for imaging applications (Fardin *et al.*, 2023 ▸), these direct detector solutions seem less adapted for full-field multi-frame imaging under high-flux white beam radiation (*i.e.* with a considerable amount of the spectral component above 20 keV as in the case when white undulator radiation is applied) (Hatsui & Graafsma, 2015 ▸; Rack *et al.*, 2010 ▸; Ruat & Ponchut, 2014 ▸).

Indirect X-ray detectors have demonstrated a higher degree of versatility with sufficient radiation hardness while having an improved spatial resolution with adequate sensitivity for high-dose imaging applications (Hartmann *et al.*, 1975 ▸). Radiation hardness of the detection arrangement can be further improved when the luminescence image emitted by a scintillator is folded away from the radiation axis via a mirror (usually 90°) and projected onto the photo-detector (Bonse & Busch, 1996 ▸). This configuration also allows the use of infinity-corrected optics in terms of magnification, which, apart from versatility and flexibility, enables pushing of the resolution limits (Koch, 1994 ▸). When used in conjunction with single-crystal scintillators and microscope objectives, these detectors can reach a spatial resolution close to the visible-light diffraction limit, while in combination with powder-based scintillators, single photon-count capabilities have been demonstrated (Koch *et al.*, 1998 ▸; Martin & Koch, 2006 ▸; Pauwels & Douissard, 2022 ▸). Consequently, indirect detectors with a folding mirror have gained widespread use in white beam imaging end-stations that utilize hard X-rays (Douissard *et al.*, 2012 ▸). Eventually, the scintillator afterglow presents a limit on the temporal resolution and the assembly needs to employ a fast-decay single-crystal scintillator if an ultra-fast visible-light-imaging camera is deployed. Such configurations rely on the use of scintillators, which have a decay time shorter than the bunch spacing in the storage ring and are used for (ultra fast) X-ray radioscopy within various arrangements (Rack *et al.*, 2013 ▸; Sinclair *et al.*, 2021 ▸; Escauriza *et al.*, 2018 ▸). Typically, the indirect detectors are well suited for phase-contrast imaging, which exploits the (partial spatial) coherence properties of the synchrotron light source. Within the most common arrangement, the imaging of the edge-enhanced modality of the object of interest is obtained by propagation, *i.e.* by leaving a propagation space between the sample and the imaging detector (Snigirev *et al.*, 1995 ▸; Cloetens *et al.*, 1996 ▸; Wilkins *et al.*, 1996 ▸). When combined with a burst-on-board ultra-fast framing sensor, such as that of the Hyper Vision HPV-X2 (Shimadzu Corp., Japan) (Kuroda *et al.*, 2016 ▸), single-bunch multiple-frame video recordings of X-ray phase-contrast radioscopy are achieved with remarkable contrast and sampling (Olbinado *et al.*, 2017 ▸). This type of optical arrangement has recently been demonstrated to have the potential to reach frame rates close to the native radio frequency of the storage ring with gigahertz sampling (Rack *et al.*, 2024 ▸).

However, with their cascade configuration, the detective quantum efficiency (DQE) of indirect detectors is driven by a wide range of parameters owing to the multiple optical components involved (Arndt & Gilmore, 1979 ▸; Koch *et al.*, 1998 ▸), inevitably leading to an overall low DQE when considering a monochromatic X-ray beam in the hard regime (Stampanoni *et al.*, 2002 ▸). This becomes a further challenge in applications that necessitate a larger numerical aperture (NA) (*i.e.* small pixel size/high optical magnification), such as in ultra-fast X-ray microscopy with intense white beam radiation comprising a significant hard spectral component (Mertens & Chawla, 2015 ▸; Riva, 2016 ▸). While the driving parameters are the stopping power and light emittance, which are dictated by the adopted scintillator material, another important parameter is the collection efficiency of the entire optical system downstream of the scintillating material (Martin *et al.*, 2009 ▸). This parameter is commonly improved by increasing the NA of the objective lens to augment the solid-angle coverage at the expense of the reduced depth of field. In addition, tuning the scintillating peak emittance wavelength (*e.g.* according to the Abbe criterion: *d* ≃ λ/2NA) can provide additional improvements. However, given the restrictions of the optical elements, such can be also achieved by adequate matching of the spectral properties of the optical components involved with the peak emittance wavelength of the scintillator. For ultra-fast and single-bunch imaging, the fast-decay LYSO (Lu_2–*x*_Y_*x*_SiO_5_:Ce) scintillator with a decay time of 40 ns and negligible afterglow is a well used single-crystal material for single-bunch imaging (Pidol *et al.*, 2004 ▸; Rutherford *et al.*, 2016 ▸), given the bunch separation of operating synchrotron storage rings [*e.g.* 4-bunch with 704 ns and 16-bunch with 176 ns at the European Synchrotron Radiation Facility (ESRF); 24-bunch with 153 ns at Advanced Photon Source]. However, more than 50% of the scintillator emitted light is within the NUV regime (*i.e.* 310–430 nm), where most of the optical microscopes have reduced spatial resolving power as well as reduced transmittance and/or reflectivity.

## 3. Indirect detector design

**Figure 1:**
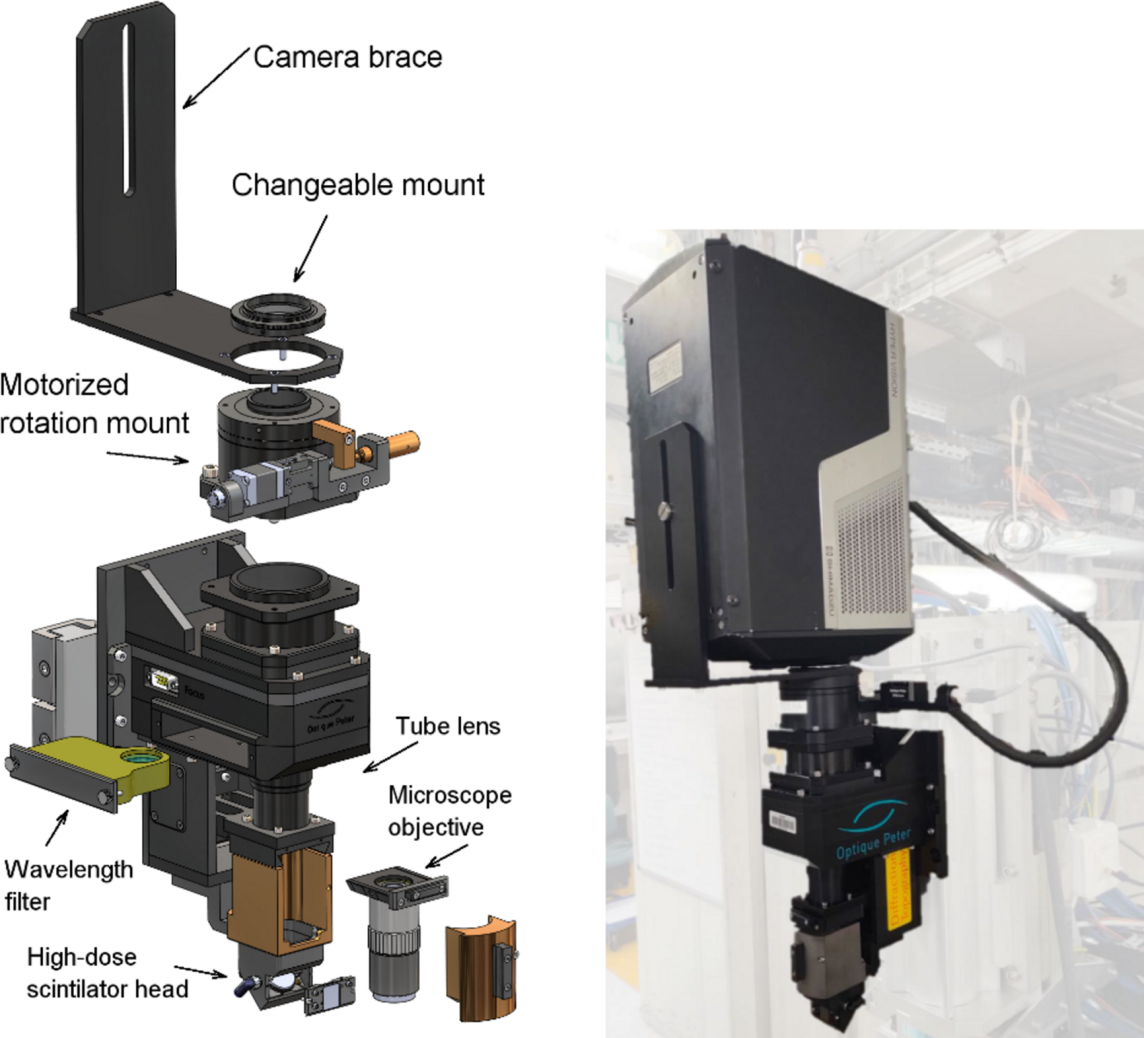
NUV detector design and assembly developed by Optique Peter. Left: a schematic representation of the modular design of the NUV detector system. Right: NUV indirect detector assembly with HPV-X2 ultra-fast camera.

The NUV-sensitive microscope follows the standardized modular design developed by Optique Peter (France) for high-dose radiation resistance, such as in the case of intense polychromatic synchrotron hard X-ray sources (Douissard *et al.*, 2012 ▸). The design accommodates a high level of compatibility, allowing for the use of commercially available optical components (Fig. 1 ▸). In its current form, the design consists of a high-dose scintillator head, a coated NUV-sensitive glassy carbon mirror, an interchangeable lens mount equipped with the NUV microscope (10× NUV M Plan APO, NA = 0.28, Mitutoyo, Japan) with 4.5 mm lead glass protection, an optional visible-light filter, a tube lens (F200 millimetre NUV/Vis, Mitutoyo, Japan) and a motorized camera rotation mount. The scintillator head consists of a spring mount system allowing for straightforward mounting and a fine manual adjustment of horizontal and vertical tilt of the scintillator in the object plane. The mirror has 23 mm diameter and 4 mm thickness with λ/10 flatness and UV-enhanced coating. The thickness of the mirror should be kept minimal to limit back-scattering and, therefore, potential darkening of the objective microscope lens, which is perpendicular to the beam path. Given the narrow depth of focus of the NUV microscope, the objective-lens mount is equipped with a micrometre-precision translation motor, allowing the objective lens to move and set the focal plane within the active layer (*i.e.* image-formation plane) within the scintillator material.

## 4. Characterization

**Figure 2:**
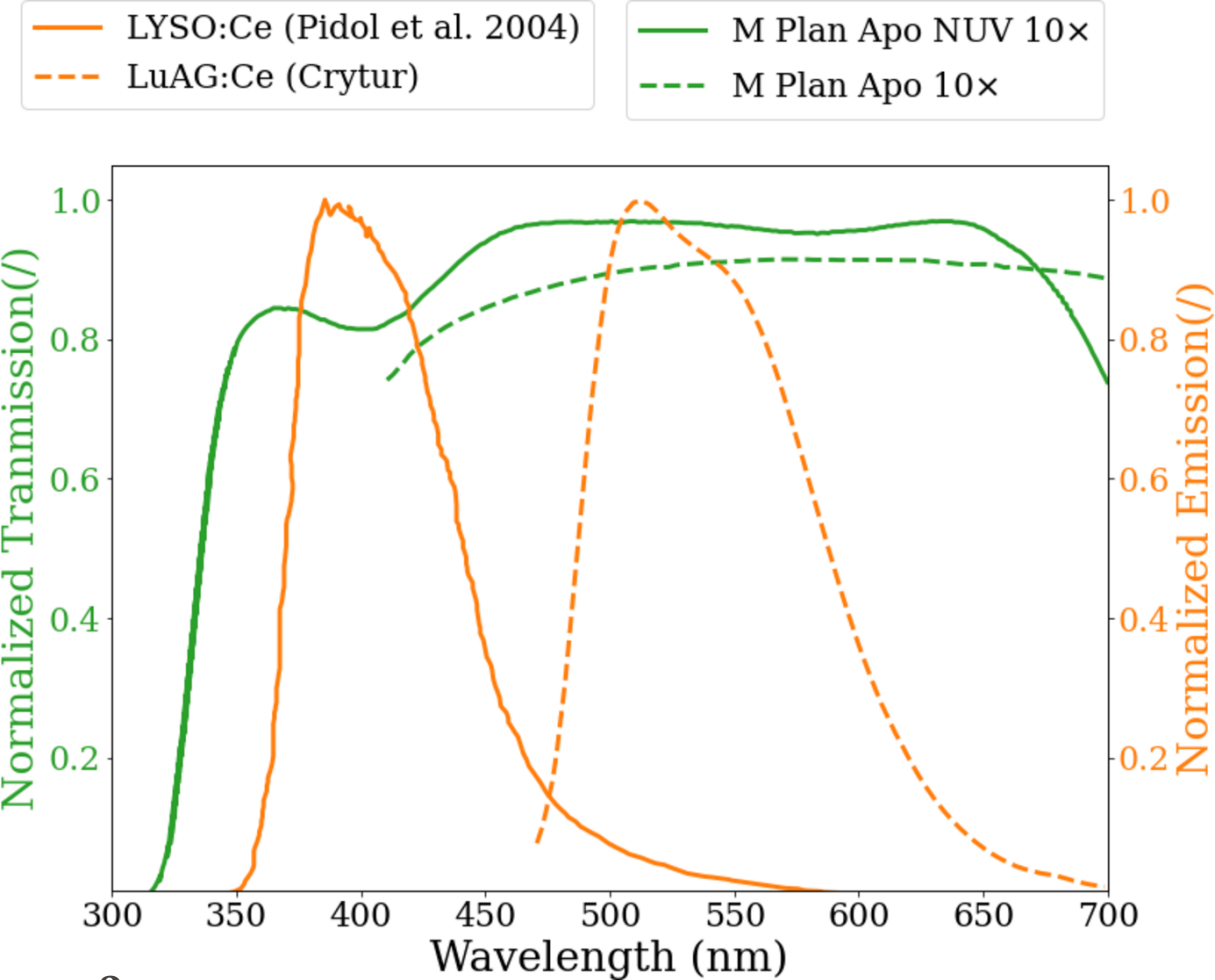
This figure depicts the benefits of component spectral matching for light-detection efficiency increase. It shows spectral transmission of the 10× NUV-sensitive and visible-light-sensitive M Plan Apo microscopes (the green curve of the 10× M Plan Apo microscope with the transmission below 400 nm is not supplied by the manufacturer). Spectral light emission is also shown for the two scintillators used: LYSO:Ce (Pidol *et al.*, 2004 ▸) and LuAG:Ce (Cryture, CZ).

In order to evaluate the benefit in terms of the overall light-collection efficiency of the NUV-sensitive microscope in contrast to standard (*i.e.* visible-light sensitive) microscopes, characterizations were performed at the ID19 beamline of ESRF (Grenoble, France). The detector assembly was exposed to a single harmonic 26 keV source (bandwidth = 1%) supplied by the U13 undulator (λ_u_ = 13 mm, total length = 1.6 m, gap = 22.5 mm) during the 200 mA storage ring current. Two configurations were considered utilizing both the LYSO:Ce (Hilger Crystals, UK) and LuAG:Ce (Crytur, CZ) 250 µm-thick scintillators, given their distinct contrast in the peak wavelength of the emission spectrum as depicted in Fig. 2 ▸. The entire detector arrangement was positioned ∼146 m away from the source. In both situations, the Hyper Vision HPV-X2 camera was utilized with the X-ray microscope with 10 ms exposure time per acquired frame. Exploiting the light from a single-harmonic undulator allows one to show the maximum performance of the indirect system in terms of spatial resolution and contrast, as no heat-load issues need to be considered, while the narrow bandwidth gives excellent phase-contrast conditions.

### 4.1. Light-collection efficiency

Acquisitions were performed by taking 128 frames with the comparison being made on a 200 × 200 pixel in the central part of the sensor, *i.e.* to show the performance of this indirect detector system compared with a detector with standard non-NUV compatible Mitutoyo lenses. The average pixel value over the entire set of frames in a single acquisition provides the amplitude of the detected signal while the standard deviation provides the overall information on the noise structure, which is expected to follow the Poisson statistic. The results are provided in Table 1 ▸. The results indicate an increase in overall efficiency of the NUV microscope, more than 2× in the case of the LYSO:Ce scintillator as well as a notable increase for the case of the LuAG:Ce scintillator, with corresponding improvement of the signal-to-noise ratio (SNR) (the increase of efficiency when using an NUV lens is not a quantitative measure here as the non-NUV lens deployed for the test has been used before and might have already been affected by darkening due to X-ray radiation). Although the effective scintillated-light-to-digital-counts conversion efficiency is 30% higher for the case of the LuAG:Ce tandem, considering the quantum efficiency of the imaging system (Kuroda *et al.*, 2016 ▸), the results indicate that the adequate spectral matching of optical components allows reaching of a comparable detected signal for the two cases.

### 4.2. X-ray spatial resolving power

While with visible-light illumination the resolution is limited by the diffraction limit of the optic (notably below the pixel size in the present case), in the case of X-ray illumination the attainable spatial resolution is limited by the diffraction limit of the objective lens and the match with the defect of focus of the adopted single-crystal scintillator (thickness) (Koch *et al.*, 1998 ▸; Stampanoni *et al.*, 2002 ▸). Adopting the approximated analytical solution provided by Koch *et al.* (1998 ▸) for full width at 50% of the integrated line spread function and assuming an inverse linear dependence of the optical transfer function with emitted wavelength, the approximated limit resolution for a 250 µm-thick scintillator is retrieved as 4.9 µm for the case of LuAG:Ce, and 3.7 µm for the case of LYSO:Ce. These approximations derived from the analytical solution provided by Hopkins (Hopkins & Burch, 1955 ▸) adopt a single-wavelength illumination, which can be considered as an approximation here.

**Figure 3:**
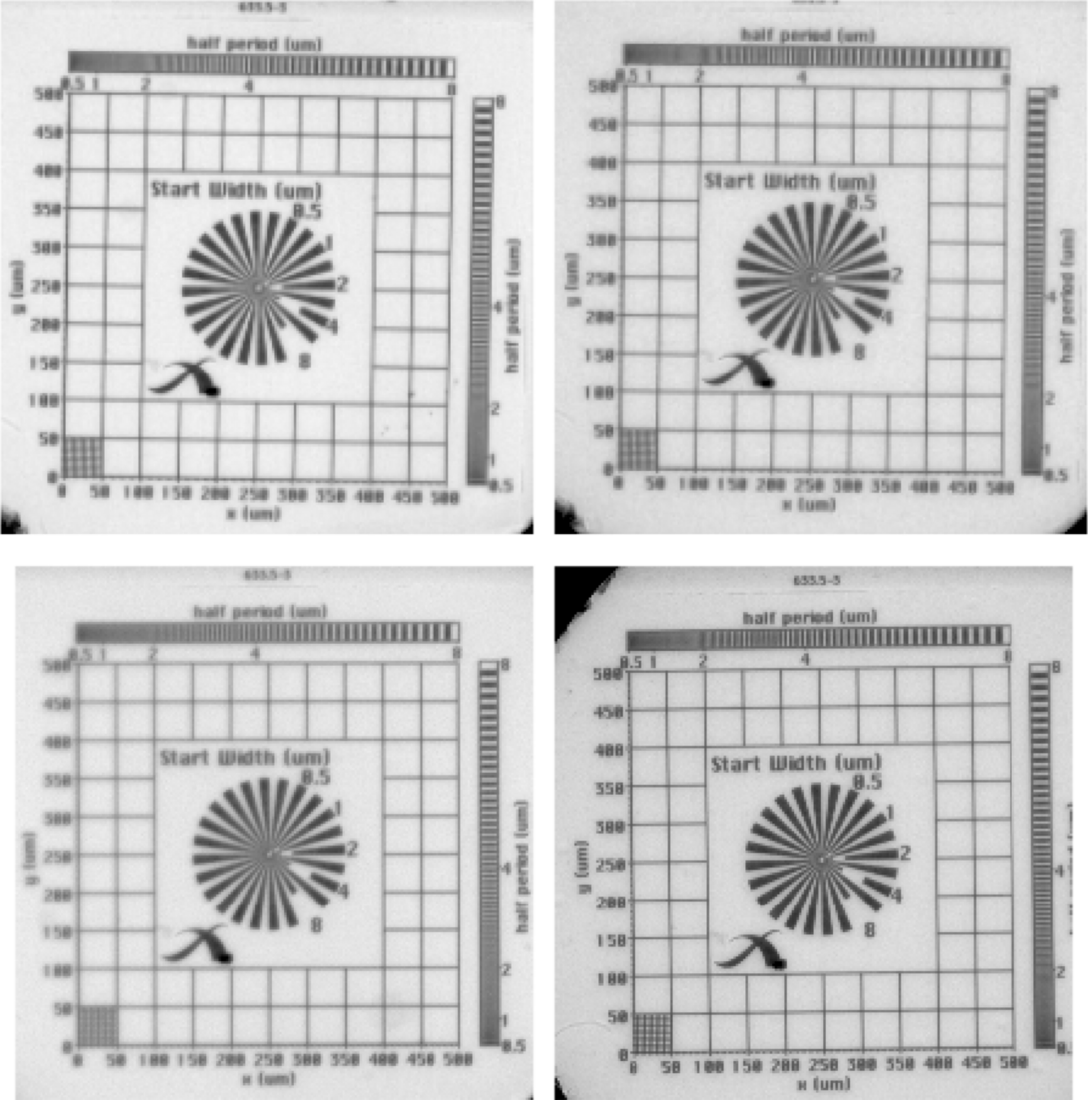
Characterization of the X-ray detector system utilizing the X-ray test pattern (Xradia Inc., model X500-200-30) and 26 keV single harmonic illumination (*t* stands for scintillator thickness). Top left: M Plan Apo with LYSO:Ce (*t* = 250 µm). Top right: M Plan Apo with LuAG:Ce (*t* = 250 µm). Bottom left: NUV M Plan Apo with LYSO:Ce (*t* = 250 µm). Bottom right: NUV M Plan Apo with LuAG:Ce (*t* = 250 µm).

**Figure 4:**
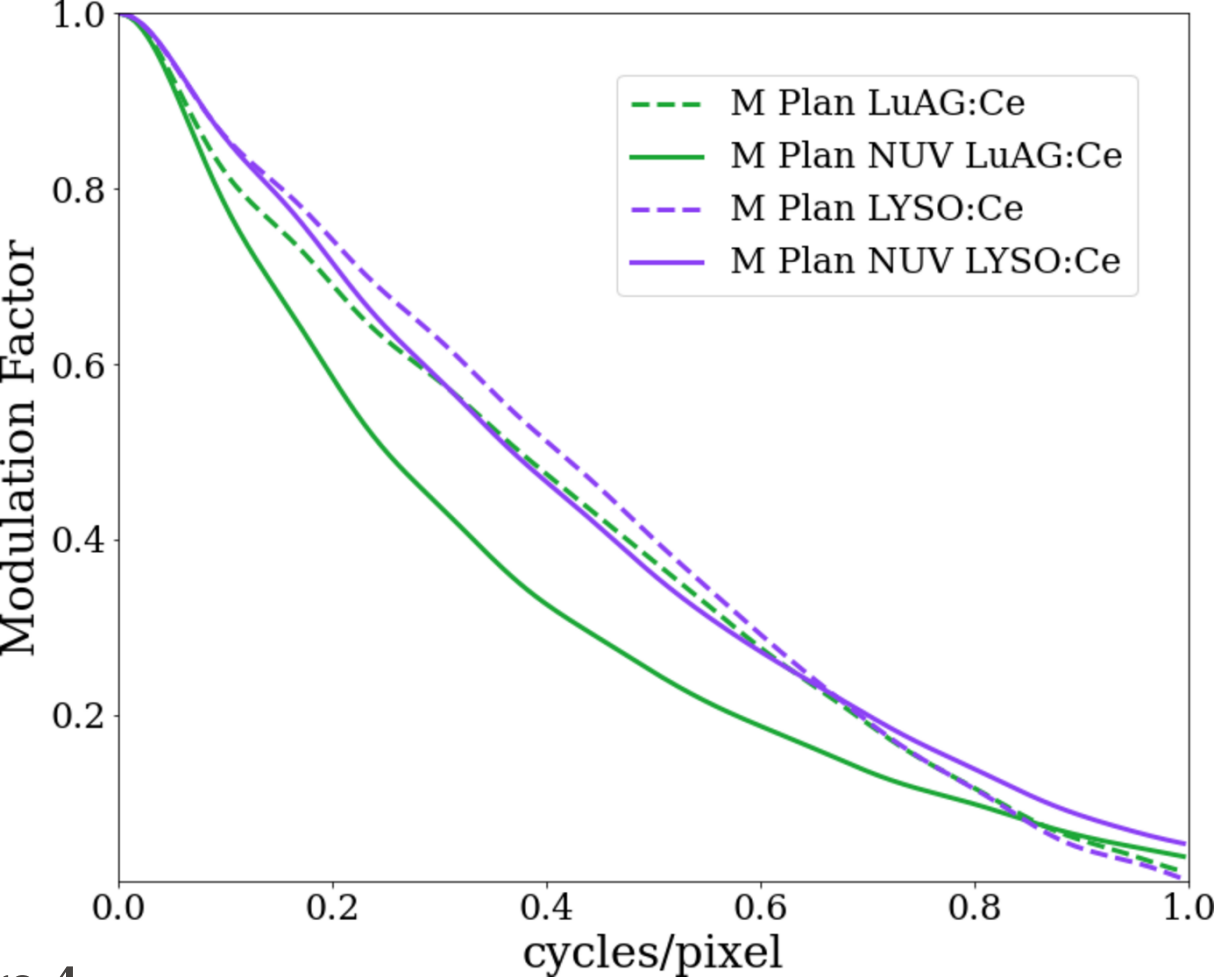
MTF obtained via the slanted-edge approach utilizing the GaAs [110] wafer along the cleavage plane inclined for 5° along the vertical axis of the detector. The cleaved edge was imaged with single harmonic X-ray illumination at 18 keV utilizing two detector arrangements; namely, with the 10× NUV-sensitive and visible-light-sensitive M Plan Apo microscopes combined with both LYSO:Ce and LuAG:Ce scintillator single crystals of 250 µm thickness.

In the present work, the resolving power of the NUV-sensitive X-ray microscope is first evaluated by performing acquisitions of the X-ray resolution test pattern (Xradia Inc., model X500-200-30) with several configurations. The test pattern is illuminated with 26 keV pink beam single harmonic, which is comparable to the median energy of the polychromatic source used for single-bunch imaging. The measurement results of the test pattern are depicted in Fig. 3 ▸ as an overall average of each acquisition considering a 250 µm-thick scintillator. The pixel size was measured to be 3.17 µm in all cases and the results suggest that in the given configuration the smallest visible feature is close to 4 µm. However, due to the limited size of the test-pattern stage mount, the contribution of propagation-based phase contrast cannot be fully avoided. In order to more closely evaluate the overall spatial resolution at the given pixel size, the modulation transfer function (MTF) is measured by adopting the slanted-edge method (Samei *et al.*, 1998 ▸). A GaAs [110] wafer is used as the slanted-edge target along the cleavage plane and positioned 10 mm away from the detector. To improve the contrast ratio by relying on the target’s attenuation contrast, the edge is illuminated with 18 keV pink beam single harmonic. The edge angle is set to ∼5° along the vertical axis of the detector, covering a length of 250 pixels. This was considered representative since the fill factor of the HPV-X2 sensor is reported to be symmetric (Kuroda *et al.*, 2016 ▸). The loss of visibility resulting from the difference in light yield is tuned by changing the undulator gap so that the contrast-to-noise ratio (CNR) remains comparable across different configurations. The results of the retrieved MTF for different detector configurations are presented in Fig. 4 ▸ and summarized in Table 2 ▸. While the slightly higher resolution of the visible-light-sensitive microscope can be explained by the higher quantum efficiency of the HPV-X2 sensor, the results clearly demonstrate the improved spatial resolution of combining the NUV-sensitive microscope with the LYSO:Ce scintillator compared with the LuAG:Ce scintillator.

## 5. Applications

**Figure 5:**
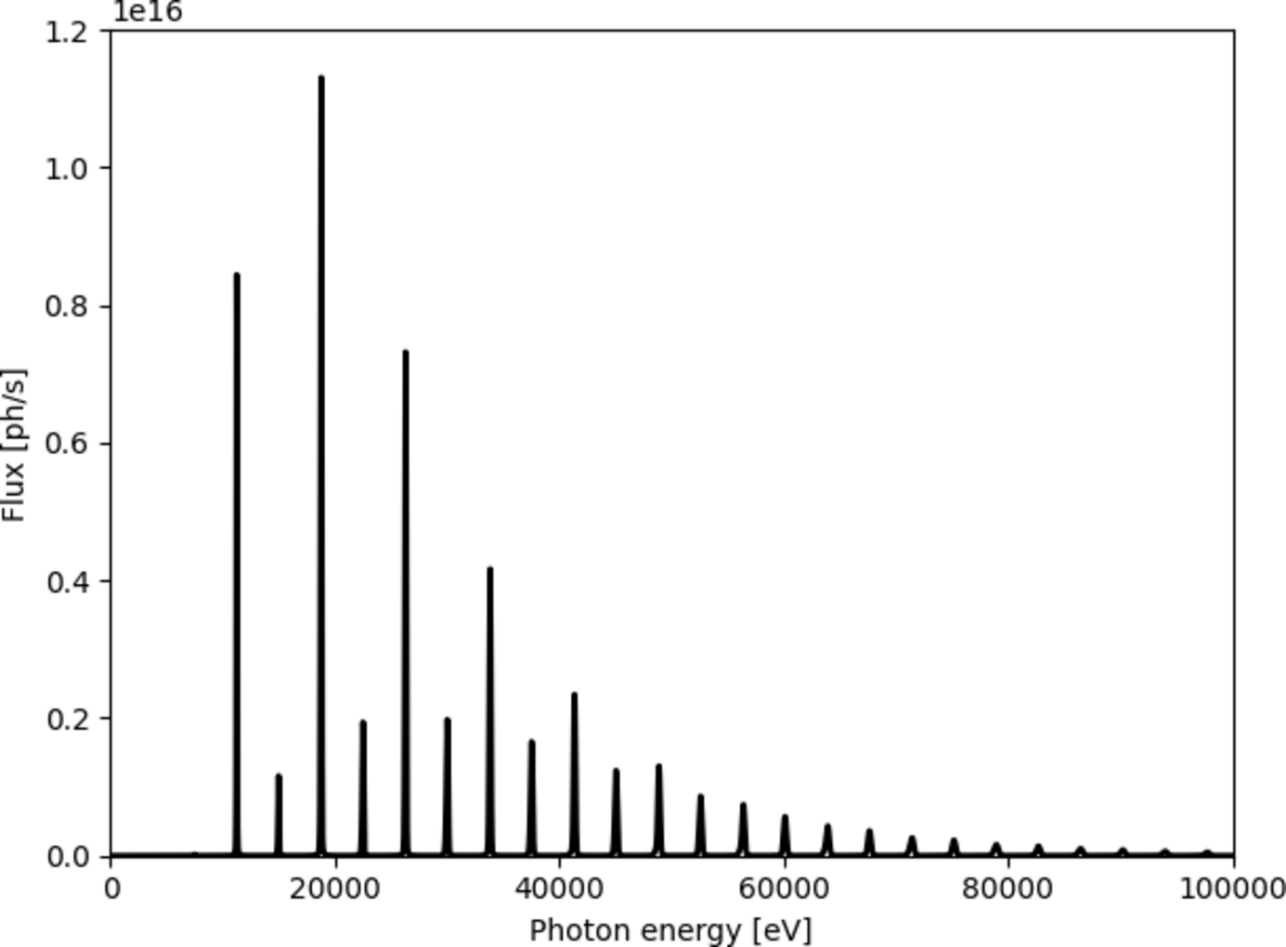
A calculated ID19 source spectrum provided by 2 × U32 devices collimated on the sample with 16 CRLs and used for single-bunch illumination (Sánchez del Río & Dejus, 2011 ▸).

Two applications of ultra-fast single-bunch X-ray micro-imaging are chosen to demonstrate the performance of the indirect detector system for real-life experiments: (1) rapid-discharge wire explosion and (2) superheated fuel injection. The experiments were carried out at the ID19 beamline of ESRF (Weitkamp *et al.*, 2010 ▸; Rack *et al.*, 2014 ▸) during the 4-bunch filling mode (10 mA per bunch). The polychromatic X-ray beam generated by two U32 undulator insertion devices (λ_u_ = 32 mm, total length = 3.2 m) set to a minimum gap of 11.5 mm was used to probe the fast observation phenomena. The generated polychromatic spectrum was filtered with mandatory optical elements along the 145 m-long vacuum flight tube (0.8 mm-thick diamond window and a series of thin carbon and beryllium windows) to reduce heat load by cutting out the soft part of the X-ray spectrum (Fig. 5 ▸). The beam was additionally collimated using the beamline’s transfocator, located 40 m downstream from the source, consisting of 16 Be-based compound refractive lenses (CRLs). The propagation distance between the sample and the indirect detector assembly was 1.2 m, which is sufficient for the propagation-based interference between transmitted X-rays to result in an increased contrast of the material’s edges while preserving the material’s geometrical representation.

### 5.1. Wire explosion

**Figure 6:**
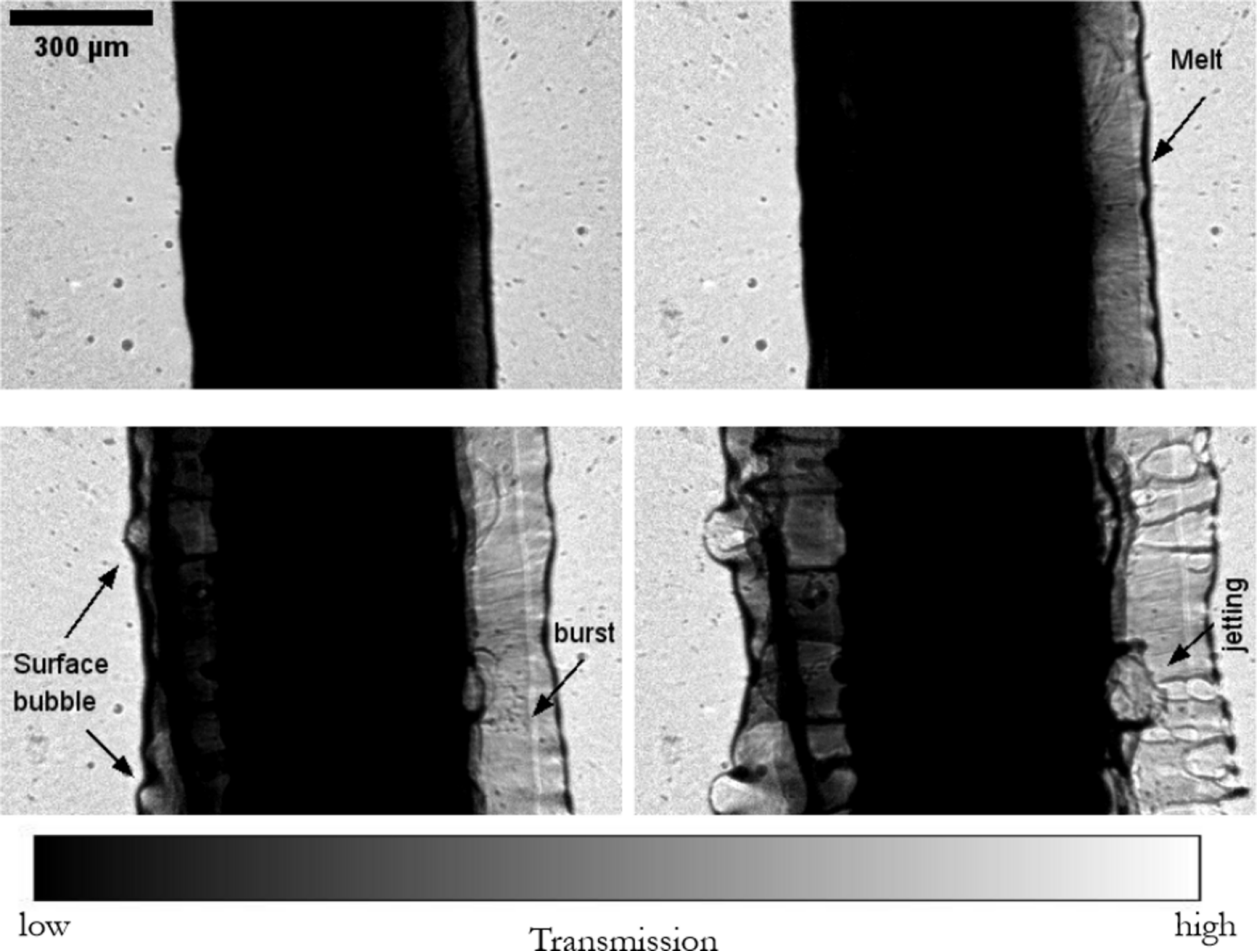
A time series of single-bunch phase-contrast radiographs of lead wire melting dynamics under sudden current discharge observed with the NUV X-ray microscope and 10× magnification (*i.e.* 3.16 µm per pixel) at 1.4 Mfps (1/710 ns). The radiographs were captured during a 4-bunch filling mode (7.5 mA per bunch). Joule heating dominates the phenomena of failure and solid-to-liquid transition of the lead wire. Top left: the onset of melting can be spotted with asymmetric expansion. Top right: the melting becomes more apparent. Bottom left: surface bubble formation and bubble burst in localized regions can be noticed. Bottom right: jet formation in a radial direction now occurs, after which complete failure occurs due to melting. (Adequate radiograph background correction was not performed due to constantly evolving background content.) Timestamps of depicted radiographs: top left = *t*_0_; top right = *t*_0_ + 7 µs; bottom left = *t*_0_ + 14 µs; and bottom right = *t*_0_ + 21 µs. The speed of the right-hand-side melt interface was calculated to be 9.7 ms^−1^.

**Figure 7:**
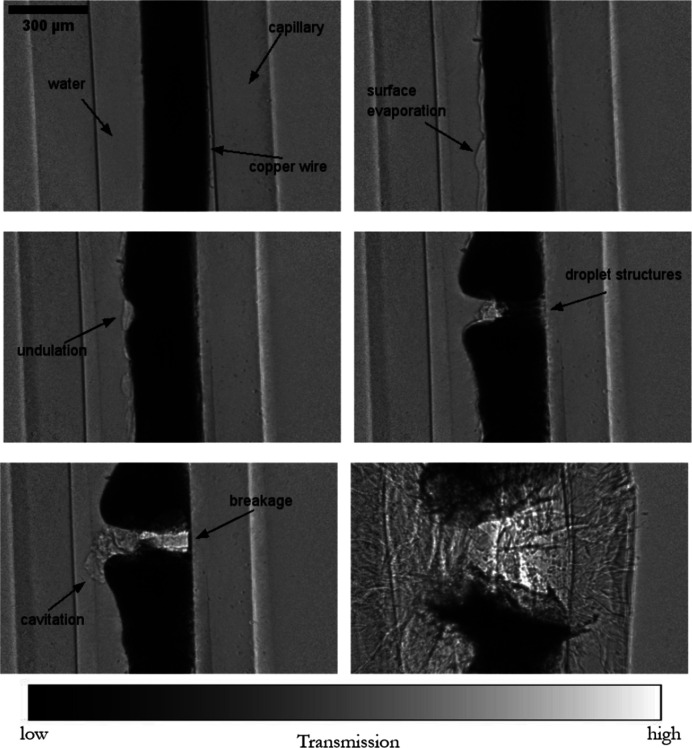
A time series of single-bunch phase-contrast radiographs of copper wire explosion in a water-filled capillary under sudden current discharge observed with the NUV X-ray microscope and 10× magnification (*i.e.* 3.16 µm per pixel) at 0.7 Mfps (1/1.42 µs). The radiographs were captured during a 4-bunch filling mode (7.5 mA per bunch). Top right: the formation of a water vaporization layer due to heating can be observed. Middle left: the onset of unduloid formation can be noticed. Middle right: this unduloid formation shows an indication of several droplet-like structures at pre-failure. Bottom left: cavitation bubble formation in surrounding water from localized failure and plasma formation can be noticed. Bottom right: the complete wire separation and fragmentation of the glass capillary due to energy discharge into the surroundings. (The radiographs are background corrected with a pseudo-flat field.) Timestamps of depicted radiographs: top left = *t*_0_; top right = *t*_0_ + 21.3 µs; middle left = *t*_0_ + 35.5 µs; middle right = *t*_0_ + 42.6 µs; bottom left = *t*_0_ + 44 µs; and bottom right = *t*_0_ + 45.4 µs.

The electrical wire explosion is a highly dynamic phenomenon that places the material and its surroundings under extreme conditions of temperature and pressure (Krasik *et al.*, 2010 ▸). These states induce strong dynamics and phase change in the materials, and, depending on the discharge densities and host medium, different primary physical phenomena are the driving mechanisms of failure during the explosion (Chace & Moore, 2014 ▸). The wire explosion has been extensively studied from both theoretical and experimental aspects, with applications ranging from the investigation of convergent underwater shock waves to rock fracturing and the production of nano-particles. However, the knowledge of the underlying physics remains relatively elusive, mostly due to experimental limitations in probing the complex multi-physics involved *in situ*. During the explosion, a sudden current discharge of high-energy density transforms rapidly into heat and kinetic energy, leading to wire expansion, metal melting and plasma formation. Spark creation often follows, as seen during arc ignition in electric fuses, making visible-light probing inadequate (De Palma & Gelet, 2017 ▸). Confined opaque environments further obscure observation of this explosive process due to the plasma creation (*e.g.* in ceramic fuse casing), making ultra-fast hard X-ray microscopy a highly suitable method to capture the full-field successive kinetics of wire failure (Olbinado *et al.*, 2017 ▸; Milliere & Lavaud, 2020 ▸). For the case of electrical fuse breaking, failure often occurs in stages: Joule heating during conduction leads towards melting, pre-arcing and then arcing, which is characterized by important phenomena of the formation of a plasma channel that can maintain the current flow. Here, two distinct examples of wire explosion are presented, initiated by a high current discharge provided by a 8.8 mF capacitor and a gate thyristor switch, reaching a peak current of 2.5 kA at 0.75 kV within 20 µs. A time series of single-bunch radiographs is depicted in Fig. 6 ▸, for a 500 µm lead wire, and in Fig. 7 ▸, for a 200 µm copper wire, as captured by the NUV X-ray microscope, marking the contrast in failure dynamics limited by differences in wire conductive properties.

### 5.2. Fuel injection dynamics

Knowledge of fuel injection dynamics is of critical importance for building efficient combustion engines (Bornschlegel *et al.*, 2021 ▸). The fluid dynamics occurring in the injector and at its exit directly affect the fuel-spray characteristics, which determine the engine efficiency and emission. Despite its importance, however, there have been only a few observations of the time-dependent structure of the fuel spray, especially in the near field of the developing spray close to the injector.

This is mainly due to the strong refraction and scattering effects occurring for radiation in the visible spectrum that render the spray completely opaque in the first 10–20 mm from the nozzle exit typically.

Sprays are ubiquitous, with extremely complex hydrodynamics occurring on small spatio-temporal scales. Spray flow is typically a multi-component, non-linear, highly turbulent, high-speed and high-pressure phenomenon and is extremely challenging to study, both theoretically and experimentally. The limitation of studying spray dynamics within visible light is limited by the fact that: (1) visible-light rays cannot penetrate regions where there are a lot of air–liquid interfaces, *e.g.* regions with large numbers of small droplets; and (2) even in regions where there can be a sufficient light transmission, it remains relatively difficult to extract quantitative information as a result of multiple scattering opportunities along light-ray paths, *i.e.* there is no longer a simple straight line-of-sight correspondence between the image and the object. While the second problem does exist with X-ray phase-contrast imaging (*i.e.* due to refraction), it is several orders of magnitude smaller compared with visible light, since the refractive index of typical liquids is 10^6^–10^7^ smaller with hard X-rays compared with visible light. Furthermore, the penetration depth of hard X-ray radiation allows observation of the intricate turbulence taking place subsurface within the injection nozzle.

**Figure 8:**
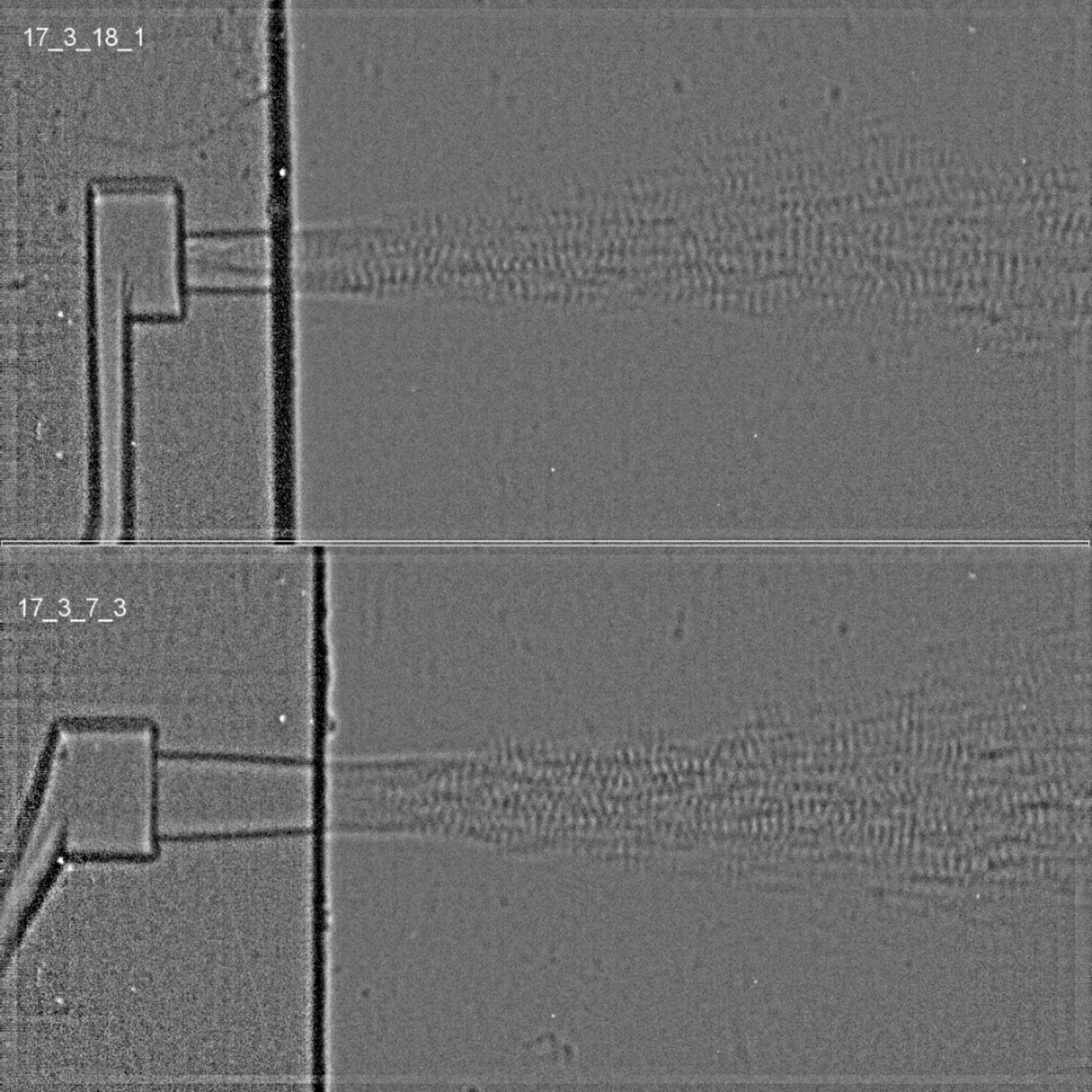
Two characteristic frames of *in situ* monitoring of fuel injection dynamics, with a perpendicular injection geometry (top) and an inclined injection geometry (bottom). The perpendicular geometry shows a more narrow jet atomization (*i.e.* spatial spread) with respect to distance from the injection nozzle in comparison with the inclined geometry. Such a spatial distribution can lead to a weak velocity gradient and an increased spray penetration, leading to wetting of the engine walls before combustion. Velocities in excess of 150 ms^−1^ in the interior of the spray have been measured using multi-bunch exposures.

An example of the multi-bunch X-ray radioscopy of the rapid fuel injection event is depicted in Fig. 8 ▸. Due to refraction and optical scattering in the visual spectrum, the near-field region close to the nozzle is opaque and techniques such as shadowgraphy cannot be used to visualize the spray structures. Real-sized quartz glass fuel nozzles with two distinct injection geometries were used, considering a converging geometry (Fig. 8 ▸, bottom) and a parallel injection geometry (Fig. 8 ▸, top) with respect to the injection direction. The NUV detector assembly was coupled with a Phantom v2640 high-speed camera (Vision Research, USA), providing an effective pixel size of 2.58 µm.

Multiple X-ray pulses (of 175 ns separation) were exposed onto a single radiograph recorded utilizing an interframe time of 31.25 µs and an LYSO:Ce scintillator, which enabled the extraction of the velocity of the ejected fluid via autocorrelation analysis (Bornschlegel *et al.*, 2018 ▸). A large field of view up to 8 mm away from the injection nozzle was covered, allowing the visualization of the spray break-up and droplet atomization in the near-field region of the injector. Qualitative analysis showed that the spray structures dispersed spatially with increasing spread for the case of the converging nozzle geometry, resulting in less spray penetration and a considerable reduction of jet speed with respect to the parallel-geometry nozzle.

## 6. Summary

This work describes the development of an optimized indirect X-ray imaging microscope specifically designed to attain exceptional performance characteristics, notably achieving micrometre pixel size and a remarkable megahertz acquisition speed. The innovation in the entire detector optical arrangement relies on enhancing the sensitivity within the NUV part of the emitted spectrum, in conjunction with single-crystal fast-decay scintillators. The characterization measurements indicated a substantial increase in overall efficiency with improved SNR. The detector configuration is well suited for high-dose applications necessitating high-flux polychromatic illumination, providing an improved spatial resolution for hard X-ray single-bunch imaging. This is exemplified through the notable examples of a pulsed wire explosion and superheated near-nozzle gasoline injection experiments. These achievements include a pixel size of 3.2 µm, acquisition rates of up to 1.4 MHz and an effective exposure time of 60 ps.

## Figures and Tables

**Table 1:** Signal measurements obtained with a 26 keV pink beam considering different detector assemblies

Objective lens	Scintillator	Detected signal (gray level)	SNR
NUV 10X M Plan Apo	LYSO:Ce	36000	29
10X M Plan Apo	LYSO:Ce	9500	15
NUV 10X M Plan Apo	LuAG:Ce	39000	31
10X M Plan Apo	LuAG:Ce	25000	20

**Table 2:** Spatial resolution measured at 50% of the modulation factor (*i.e.* MTF-50) obtained from MTF curves from Fig. 4 ▸ with detector pixel size measured to be 3.17 µm

Objective lens	Scintillator	Resolution (pixels)	CNR
NUV 10X M Plan Apo	LYSO:Ce	2.72	170
10X M Plan Apo	LYSO:Ce	2.43	150
NUV 10X M Plan Apo	LuAG:Ce	4	111
10X M Plan Apo	LuAG:Ce	2.67	150

## References

[bb1] Allahgholi, A., Becker, J., Bianco, L., Delfs, A., Dinapoli, R., Goettlicher, P., Graafsma, H., Greiffenberg, D., Hirsemann, H., Jack, S., Klanner, R., Klyuev, A., Krueger, H., Lange, S., Marras, A., Mezza, D., Mozzanica, A., Rah, S., Xia, Q., Schmitt, B., Schwandt, J., Sheviakov, I., Shi, X., Smoljanin, S., Trunk, U., Zhang, J. & Zimmer, M. (2015). *J. Instrum.* **10**, C01023.

[bb2] Arndt, U. W. & Gilmore, D. J. (1979). *J. Appl. Cryst.* **12**, 1–9.

[bb3] Barna, S., Shepherd, J., Tate, M., Wixted, R., Eikenberry, E. & Gruner, S. (1997). *IEEE Trans. Nucl. Sci.* **44**, 950–956.

[bb4] Bokman, G. T., Biasiori-Poulanges, L., Lukić, B., Bourquard, C., Meyer, D. W., Rack, A. & Supponen, O. (2023). *Phys. Fluids*, **35**, 013322.

[bb5] Bonse, U. & Busch, F. (1996). *Prog. Biophys. Mol. Biol.* **65**, 133–169. 10.1016/s0079-6107(96)00011-9 9029944

[bb6] Bornschlegel, S., Conrad, C., Durst, A., Wang, J. & Wensing, M. (2018). *Int. J. Eng. Res.* **19**, 67–77.

[bb7] Bornschlegel, S., Conrad, C., Durst, A., Welss, R., Wensing, M., Olbinado, M., Helfen, L. & Baumbach, T. (2021). *Int. J. Eng. Res.* **22**, 592–605.

[bb8] Cammarata, M., Eybert, L., Ewald, F., Reichenbach, W., Wulff, M., Anfinrud, P., Schotte, F., Plech, A., Kong, Q., Lorenc, M., Lindenau, B., Räbiger, J. & Polachowski, S. (2009). *Rev. Sci. Instrum.* **80**, 015101. 10.1063/1.3036983 19191457

[bb9] Chace, W. G. & Moore, H. K. (2014). *Exploding Wires*, Vol. 4. Springer.

[bb10] Cloetens, P., Barrett, R., Baruchel, J., Guigay, J.-P. & Schlenker, M. (1996). *J. Phys. D Appl. Phys.* **29**, 133–146.

[bb11] De Palma, J.-F. & Gelet, J.-L. (2017). *Proceedings of the 2017 19th European Conference on Power Electronics and Applications (EPE’17 ECCE Europe)*, 11–14 September 2017, Warsaw, Poland. IEEE.

[bb12] Douissard, P.-A., Cecilia, A., Rochet, X., Chapel, X., Martin, T., Kamp, T., Helfen, L., Baumbach, T., Luquot, L., Xiao, X., Meinhardt, J. & Rack, A. (2012). *J. Instrum.* **7**, P09016.

[bb13] Driel, T. B. van, Nelson, S., Armenta, R., Blaj, G., Boo, S., Boutet, S., Doering, D., Dragone, A., Hart, P., Haller, G., Kenney, C., Kwaitowski, M., Manger, L., McKelvey, M., Nakahara, K., Oriunno, M., Sato, T. & Weaver, M. (2020). *J. Synchrotron Rad.* **27**, 608–615. 10.1107/S1600577520004257 PMC7206547 32381760

[bb14] Escauriza, E. M., Duarte, J. P., Chapman, D. J., Rutherford, M. E., Farbaniec, L., Jonsson, J. C., Smith, L. C., Olbinado, M. P., Skidmore, J., Foster, P., Ringrose, T., Rack, A. & Eakins, D. E. (2020). *Sci. Rep.* **10**, 8455. 10.1038/s41598-020-64669-y PMC7242352 32439927

[bb15] Escauriza, E. M., Olbinado, M. P., Rutherford, M. E., Chapman, D. J., Jonsson, J. C. Z., Rack, A. & Eakins, D. E. (2018). *Appl. Opt.* **57**, 5004–5010. 10.1364/AO.57.005004 30117959

[bb16] Fardin, L., Giaccaglia, C., Busca, P. & Bravin, A. (2023). *Phys. Med.* **108**, 102571. 10.1016/j.ejmp.2023.102571 36989977

[bb17] Fezzaa, K. & Wang, Y. (2008). *Phys. Rev. Lett.* **100**, 104501. 10.1103/PhysRevLett.100.104501 18352193

[bb18] Gruner, S. M., Tate, M. W. & Eikenberry, E. F. (2002). *Rev. Sci. Instrum.* **73**, 2815–2842.

[bb19] Hart, P. A., Carpenter, A., Claus, L., Damiani, D., Dayton, M., Decker, F.-J., Gleason, A., Heimann, P., Hurd, E., McBride, E., Nelson, S., Sanchez, M., Song, S. & Zhu, D. (2019). *Proc. SPIE*, **11038**, 110380Q.

[bb20] Hartmann, W., Markewitz, G., Rettenmaier, U. & Queisser, H. J. (1975). *Appl. Phys. Lett.* **27**, 308–309.

[bb21] Hatsui, T. & Graafsma, H. (2015). *IUCrJ*, **2**, 371–383. 10.1107/S205225251500010X PMC4420547 25995846

[bb22] Hodge, D. S., Leong, A. F. T., Pandolfi, S., Kurzer-Ogul, K., Montgomery, D. S., Aluie, H., Bolme, C., Carver, T., Cunningham, E., Curry, C. B., Dayton, M., Decker, F.-J., Galtier, E., Hart, P., Khaghani, D., Ja Lee, H., Li, K., Liu, Y., Ramos, K., Shang, J., Vetter, S., Nagler, B., Sandberg, R. L. & Gleason, A. E. (2022). *Opt. Express*, **30**, 38405–38422. 10.1364/OE.472275 36258406

[bb23] Hopkins, H. H. & Burch, C. R. (1955). *Proc. R. Soc. London Ser. A*, **231**, 91–103.

[bb24] Hudspeth, M., Claus, B., Dubelman, S., Black, J., Mondal, A., Parab, N., Funnell, C., Hai, F., Qi, M., Fezzaa, K., Luo, S. N. & Chen, W. (2013). *Rev. Sci. Instrum.* **84**, 025102. 10.1063/1.4789780 23464246

[bb25] Katagiri, K., Pikuz, T., Fang, L., Albertazzi, B., Egashira, S., Inubushi, Y., Kamimura, G., Kodama, R., Koenig, M., Kozioziemski, B., Masaoka, G., Miyanishi, K., Nakamura, H., Ota, M., Rigon, G., Sakawa, Y., Sano, T., Schoofs, F., Smith, Z. J., Sueda, K., Togashi, T., Vinci, T., Wang, Y., Yabashi, M., Yabuuchi, T., Dresselhaus-Marais, L. E. & Ozaki, N. (2023). *Science*, **382**, 69–72. 10.1126/science.adh5563 37796999

[bb26] Koch, A. (1994). *Nucl. Instrum. Methods Phys. Res. A*, **348**, 654–658.

[bb27] Koch, A., Raven, C., Spanne, P. & Snigirev, A. (1998). *J. Opt. Soc. Am. A*, **15**, 1940–1951.

[bb28] Krasik, Y. E., Fedotov, A., Sheftman, D., Efimov, S., Sayapin, A., Gurovich, V. T., Veksler, D., Bazalitski, G., Gleizer, S., Grinenko, A. & Oreshkin, V. I. (2010). *Plasma Sources Sci. Technol.* **19**, 034020.

[bb29] Kuroda, R., Tochigi, Y., Miyauchi, K., Takeda, T., Sugo, H., Shao, F. & Sugawa, S. (2016). *ITE Trans. Media Technol. Applic.* **4**, 149–154.

[bb30] Lee, J. S., Weon, B. M., Park, S. J., Je, J. H., Fezzaa, K. & Lee, W.-K. (2011). *Nat. Commun.* **2**, 367. 10.1038/ncomms1369 PMC3156824 21694715

[bb31] Lukić, B., Saletti, D., Forquin, P., Blasone, M., Cohen, A. & Rack, A. (2024). *J. Dyn. Behav. Mater.* **10**, 124–136.

[bb32] Maler, D., Efimov, S., Liverts, M., Theocharous, S., Strucka, J., Yao, Y., Proud, W., Rack, A., Lukic, B., Bland, S. N. & Krasik, Y. E. (2022). *Phys. Plasmas*, **29**, 063502.

[bb33] Martin, T., Douissard, P.-A., Couchaud, M., Cecilia, A., Baumbach, T., Dupre, K. & Rack, A. (2009). *IEEE Trans. Nucl. Sci.* **56**, 1412–1418.

[bb34] Martin, T. & Koch, A. (2006). *J. Synchrotron Rad.* **13**, 180–194. 10.1107/S0909049506000550 16495618

[bb35] Mertens, J. C. & Chawla, N. (2015). *Nucl. Instrum. Methods Phys. Res. A*, **783**, 110–116.

[bb36] Milliere, L. & Lavaud, P. (2020). *Plasma Res. Express*, **2**, 035005.

[bb37] Montgomery, D. S. (2023). *Rev. Sci. Instrum.* **94**, 021103. 10.1063/5.0127497 36859012

[bb38] Olbinado, M. P., Just, X., Gelet, J.-L., Lhuissier, P., Scheel, M., Vagovic, P., Sato, T., Graceffa, R., Schulz, J., Mancuso, A., Morse, J. & Rack, A. (2017). *Opt. Express*, **25**, 13857–13871. 10.1364/OE.25.013857 28788829

[bb39] Pauwels, K. & Douissard, P.-A. (2022). *J. Synchrotron Rad.* **29**, 1394–1406. 10.1107/S1600577522009584 PMC9641558 36345747

[bb40] Philipp, H. T., Tate, M. W., Purohit, P., Shanks, K. S., Weiss, J. T. & Gruner, S. M. (2016). *J. Synchrotron Rad.* **23**, 395–403. 10.1107/S1600577515022754 PMC4768764 26917125

[bb41] Pidol, L., Kahn-Harari, A., Viana, B., Virey, E., Ferrand, B., Dorenbos, P., de Haas, J. & van Eijk, C. (2004). *IEEE Trans. Nucl. Sci.* **51**, 1084–1087.

[bb42] Pradel, P., de Rességuier, T., Malaise, F., Olbinado, M. P., Rack, A., Grenzer, J., Loison, D. & Berthe, L. (2022). *J. Appl. Phys.* **131**, 055106.

[bb43] Rack, A. (2020). *Synchrotron Radiat. News*, **33**(3), 20–28.

[bb44] Rack, A., García-Moreno, F., Helfen, L., Mukherjee, M., Jiménez, C., Rack, T., Cloetens, P. & Banhart, J. (2013). *Appl. Opt.* **52**, 8122–8127. 10.1364/AO.52.008122 24513767

[bb45] Rack, A., Garcia-Moreno, F., Schmitt, C., Betz, O., Cecilia, A., Ershov, A., Rack, T., Banhart, J. & Zabler, S. (2010). *J. X-ray Sci. Technol.* **18**, 429. 10.3233/XST-2010-0273 21045279

[bb46] Rack, A., Scheel, M., Hardy, L., Curfs, C., Bonnin, A. & Reichert, H. (2014). *J. Synchrotron Rad.* **21**, 815–818. 10.1107/S1600577514005852 PMC4073960 24971980

[bb47] Rack, A., Sekiguchi, H., Uesugi, K., Yasuda, N., Takano, Y., Okinaka, T., Iguchi, A., Milliere, L., Lukić, B., Olbinado, M. P. & Etoh, T. G. (2024). *Nucl. Instrum. Methods Phys. Res. A*, **1058**, 168812.

[bb48] Raimondi, P., Benabderrahmane, C., Berkvens, P., Biasci, J. C., Borowiec, P., Bouteille, J.-F., Brochard, T., Brookes, N. B., Carmignani, N., Carver, L. R., Chaize, J.-M., Chavanne, J., Checchia, S., Chushkin, Y., Cianciosi, F., Di Michiel, M., Dimper, R., D’Elia, A., Einfeld, D., Ewald, F., Farvacque, L., Goirand, L., Hardy, L., Jacob, J., Jolly, L., Krisch, M., Le Bec, G., Leconte, I., Liuzzo, S. M., Maccarrone, C., Marchial, T., Martin, D., Mezouar, M., Nevo, C., Perron, T., Plouviez, E., Reichert, H., Renaud, P., Revol, J.-L., Roche, B., Scheidt, K.-B., Serriere, V., Sette, F., Susini, J., Torino, L., Versteegen, R., White, S. & Zontone, F. (2023). *Commun. Phys.* **6**, 82.

[bb50] Riva, F. (2016). *Development of new thin film scintillators for high-resolution X-ray imaging.* PhD Thesis, Université de Lyon, France.

[bb51] Ruat, M. & Ponchut, C. (2014). *J. Instrum.* **9**, C04030.

[bb52] Rutherford, M. E., Chapman, D. J., White, T. G., Drakopoulos, M., Rack, A. & Eakins, D. E. (2016). *J. Synchrotron Rad.* **23**, 685–693. 10.1107/S1600577516002770 PMC4853870 27140147

[bb53] Samei, E., Flynn, M. J. & Reimann, D. A. (1998). *Med. Phys.* **25**, 102–113. 10.1118/1.598165 9472832

[bb49] Sánchez del Río, M. & Dejus, R. J. (2011). *Proc. SPIE*, **8141**, 814115.

[bb54] Schropp, A., Hoppe, R., Meier, V., Patommel, J., Seiboth, F., Ping, Y., Hicks, D. G., Beckwith, M. A., Collins, G. W., Higginbotham, A., Wark, J. S., Lee, H. J., Nagler, B., Galtier, E. C., Arnold, B., Zastrau, U., Hastings, J. B. & Schroer, C. G. (2015). *Sci. Rep.* **5**, 11089. 10.1038/srep11089 PMC4650669 26086176

[bb55] Sinclair, N. W., Turneaure, S. J., Wang, Y., Zimmerman, K. & Gupta, Y. M. (2021). *J. Synchrotron Rad.* **28**, 1216–1228. 10.1107/S1600577521003775 34212887

[bb56] Snigirev, A., Snigireva, I., Kohn, V., Kuznetsov, S. & Schelokov, I. (1995). *Rev. Sci. Instrum.* **66**, 5486–5492.

[bb57] Stampanoni, M., Borchert, G., Wyss, P., Abela, R., Patterson, B., Hunt, S., Vermeulen, D. & Rüegsegger, P. (2002). *Nucl. Instrum. Methods Phys. Res. A*, **491**, 291–301.

[bb58] Strucka, J., Lukic, B., Koerner, M., Halliday, J. W. D., Yao, Y., Mughal, K., Maler, D., Efimov, S., Skidmore, J., Rack, A., Krasik, Y., Chittenden, J. & Bland, S. N. (2023). *Phys. Fluids*, **35**, 044108.

[bb59] Wang, Y., Liu, X., Im, K.-S., Lee, W.-K., Wang, J., Fezzaa, K., Hung, D. L. S. & Winkelman, J. R. (2008). *Nat. Phys.* **4**, 305–309.

[bb60] Wang, Z., Leong, A. F., Dragone, A., Gleason, A. E., Ballabriga, R., Campbell, C., Campbell, M., Clark, S. J., Da Vià, C., Dattelbaum, D. M., Demarteau, M., Fabris, L., Fezzaa, K., Fossum, E. R., Gruner, S. M., Hufnagel, T., Ju, X., Li, K., Llopart, X., Lukić, B., Rack, A., Strehlow, J., Therrien, A. C., Thom-Levy, J., Wang, F., Xiao, T., Xu, M. & Yue, X. (2023). *Nucl. Instrum. Methods Phys. Res. A*, **1057**, 168690.

[bb61] Weitkamp, T., Tafforeau, P., Boller, E., Cloetens, P., Valade, J.-P., Bernard, P., Peyrin, F., Ludwig, W., Helfen, L., Baruchel, J., Garrett, R., Gentle, I., Nugent, K. & Wilkins, S. (2010). *AIP Conf. Proc.* **1234**, 83–86.

[bb62] Wilkins, S. W., Gureyev, T. E., Gao, D., Pogany, A. & Stevenson, A. W. (1996). *Nature*, **384**, 335–338.

[bb63] Zielinski, B., Sadat, T., Lukić, B., Haugou, G., Morvan, H., Rack, A., Markiewicz, E. & Dubar, L. (2023). *Mater. Lett.* **337**, 133943.

